# Parameters of the Earth’s Free Core Nutation from Diurnal Strain Tides

**DOI:** 10.1038/s41598-020-66426-7

**Published:** 2020-06-16

**Authors:** Antonella Amoruso, Luca Crescentini

**Affiliations:** University of Salerno, Department of Physics, Fisciano, 84084 Italy

**Keywords:** Core processes, Geodynamics, Geophysics

## Abstract

Earth deformation at the diurnal tidal frequencies includes the resonant tidal-forcing response caused by the Free Core Nutation (FCN), a retrograde mode related to the slight misalignment of the rotation axes of the outer core and mantle. We analyse data from four underground high-sensitivity laser extensometers, whose signal-to-noise ratio in the diurnal tidal band is particularly high, and provide an alternative independent estimate of the FCN complex frequency with respect to more usual techniques (nutation and gravity). Firstly, we differentiate displacements due to diurnal solid tides to obtain extension along any azimuthal direction in terms of three complex parameters (A, S, C) which depend on latitude and frequency. Then, we demonstrate that we can invert the FCN complex frequency and the sensitivity of Im(A) and Re(S) to the resonance from our data. Lastly we obtain the probability distributions of those four parameters. Our results are in full agreement with those from nutation and gravity, as well as with reference IERS (International Earth Rotation and Reference Systems Service) values. Sensitivities of Im(A) and Re(S) to the resonance are estimated here for the first time and are in agreement with values computed using reference Love and Shida numbers from IERS.

## Introduction

The Free Core Nutation (FCN) is a retrograde mode related to the slight misalignment of the rotation axes of the Earh’s fluid outer core and mantle, with a period *T*_*FCN*_ ∼ 430 sidereal days in the celestial reference frame. It is influenced by the flattening of the core-mantle boundary and by any deformations on the surface of that boundary. These deformations can be induced by various mechanisms: among the most relevant, there are the fluid pressure on the outer core-mantle boundary (CMB) and the electromagnetic torque due to the differential rotation of the outer core and mantle in the presence of magnetic fields and visco-magnetic friction at the CMB, which leads to a partly dissipative coupling. The FCN causes resonance effects on the forced nutations of the Earth’s figure axis^1^.

In the Earth-fixed reference frame, the FCN complex frequency is *f*_*FCN*_ = (1 + 1/*T*_*FCN*_) (1 + i/(2*Q*_*FCN*_)) cycles per sidereal day (cpsd), where *Q*_*FCN*_ is the quality factor of the resonance. Since *f*_*FCN*_ falls inside the diurnal tidal band, the FCN also causes a resonant response of some diurnal tides (e. g. P_1_, K_1_, ${\Psi }_{1}$ and ${\Phi }_{1}$) to the tide-generating forces. Gravity, displacement, strain and tilt tides can be expressed in terms of the Love (*h*) and Shida (*l*) numbers, which characterize the Earth response to the tide-generating forces, so that the FCN can be described through the resonant behaviour of *h* and *l* in the diurnal band (for a general summary, see, e.g.^2^). Diurnal Love and Shida numbers are usually given as a function of the tidal excitation frequency *σ* by the resonance formula (e.g.^3^)

1 $$L(\sigma )={L}_{0}+\mathop{\sum }\limits_{j=1}^{3}\frac{{L}_{j}}{(\sigma -{\sigma }_{j})},$$

where *σ*_*j*_ are the resonance frequencies associated with the Chandler wobble (CW, *j* = 1), the FCN (j = 2), and the free inner core nutation (FICN, *j* = 3), *σ* and *σ*_*j*_ are expressed in cpsd, and all the parameters are complex. The International Earth Rotation and Reference Systems Service (IERS) Conventions (2010)^3^ adopted reference CW, FCN, and FICN resonance frequencies from^4^; in particular, ${\sigma }_{2}={f}_{FCN}\mathrm{=1.0023181}+0.000025{\rm{i}}$. A fourth free rotational frequency of the Earth (the inner core wobble^5^) is not in Eq. 1 because its resonant frequency (about 0.0004 cpsd) is very far from the diurnal tidal band and its effects on diurnal tides are negligible. Love and Shida numbers depend on latitude, because rotation and ellipticity couple the spheroidal deformation of a given degree to spherical harmonics of other degrees, and spheroidal to toroidal modes of deformation.

Investigation of the resonant modifications of diurnal tides and forced nutations sheds light on the FCN, its related resonance parameters, the acting physical mechanisms, the relevant Earth geophysical properties, and ultimately the Earth interior. Many different research and application fields (e.g., the Earth’s orientation in the space and global reference systems) would benefit of an accurate determination of the FCN parameters. FCN effects on gravity tides (measured by gravimeters) and forced nutations (observed by very long baseline interferometry, VLBI) have been used since decades to estimate the FCN complex frequency (see e.g.^4,6–11^). VLBI observations also provide celestial pole offset (CPO) series (i. e., the difference between the observed celestial pole position and the one obtained from conventional precession and nutation models^3^) which include a FCN term. Empirical FCN models give temporal variations of the FCN term amplitude and phase, mainly related to geomagnetic jerks and atmospheric and oceanic excitations (e.g.^12–16^); temporal variations (few sidereal days) of *T*_*FCN*_ have also been suggested (e.g.^17,18^). Some recent estimates of *T*_*FCN*_ and *Q*_*FCN*_ are listed in Table 1.

**Table 1:** Estimated FCN parameters and comparison with some recent results from the analysis of VLBI and gravity data.

Author	Data	*T*_*FCN*_	*Q*_*FCN*_
Rosat *et al*.^10^	gravity	429 ± 3^(a)^	7,762 to 31,989^(b)^
Krásná *et al*.^12^	VLBI	431.18 ± 0.10^(a)^	—
Chao & Hsieh^53^	VLBI	441 ± 4.5^(c)^	—
Vondrák & Ron^15^	VLBI	431.46 ± 0.04^(a)^	19,500 ± 200^(a)^
Rosat *et al*.^11^	VLBI	430.48 to 431.28^(d)^	15,392 to 16,866^(d)^
Rosat *et al*.^11^	gravity	397 to 516^(d)^	7,763 to 323,888^(d)^
Nurul Huda *et al*.^16^ ^(e)^	VLBI	431.33 ± 0.15^(a)^	$17,{346}_{-414}^{+395}$^(f)^
this work^(g)^	strain	429.1 (420.4 to 438.0)	11,492 (3,392 to 7.2 × 10^7^)

*T*_*FCN*_ is in sideral days. Notes: ^(a)^Formal error; ^(b)^90% confidence interval; ^(c)^1σ from Monte Carlo simulations; ^(d)^95% confidence interval; ^(e)^Mean values between the complete direct and indirect procedures, as obtained from Table 4 in;^16^ ^(f)^ from ${\log }_{10}({Q}_{FCN})$ formal error; ^(g)^Median and 95% confidence interval, as obtained after stacking histograms in Fig. 4c.

**Figure 1:**
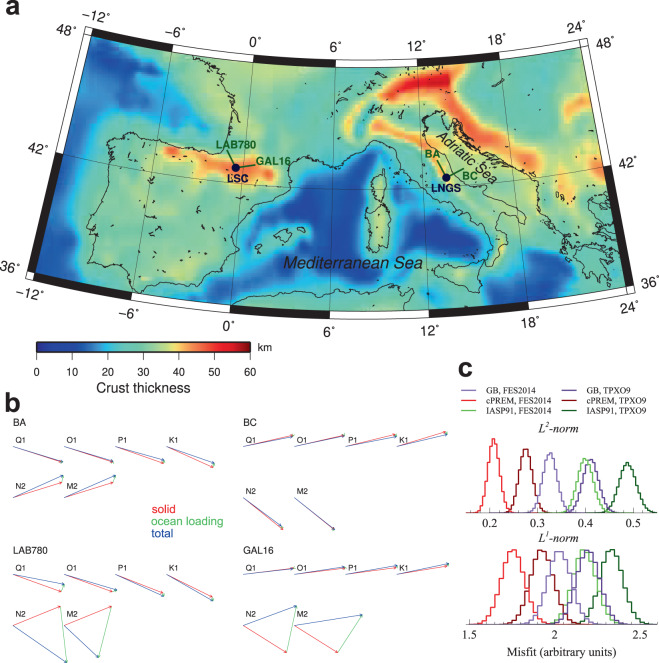
Ocean loading effects on strain tides. (**a**) Crustal thickness (interpolated from the 15′ × 15′ EuCRUST-07 model^52^) of Southern Europe (map generated using GMT v. 4.5.12, https://www.generic-mapping-tools.org/); locations (solid blue circles) of the LNGS (Laboratori Nazionali del Gran Sasso) and LSC (Laboratorio Subterráneo de Canfranc) laboratories and directions (solid green lines) of the two pairs of laser geodetic strainmeters (BA, BC and LAB780, GAL16) are also shown. (**b**) Phasor plot of solid tides (red arrows), load tides (green arrows) and total tides (blue arrows) computed along the interferometers. Amplitudes are normalized to the solid components. As for phasor directions, a horizontal rightward arrow would indicate 0° phase lag with respect to local potential; counterclockwise angles indicate positive phase lags. Solid tides are computed using Eqs. 4 and 5 and the reference IERS 2010^3^ frequency-dependent Love and Shida numbers; load tides are computed using FES2014^34^ ocean model and cPREM^36^ Earth model. Results are similar if the other ocean and Earth models are used (see Data and Methods). (**c**) Misfit distributions (arbitrary units) obtained when using different Earth and ocean models for computing ocean loading.

Few observations of the FCN have been made using borehole water level, tilt and/or strain^19–26^. In this paper we use extension data (fractional change of instrumental length, i.e., linear strain) from two pairs of underground high-sensitivity laser strainmeters (interferometers, Fig. 1a, see Data and Methods for details); strainmeters and extensometers are often used as synonyms in the literature. Two strainmeters (BA and BC in what follows) were located inside the Gran Sasso underground laboratories (LNGS, Laboratori Nazionali del Gran Sasso, Italy)^27^. The other two strainmeters (LAB780 and GAL16 in what follows) are located close to the Canfranc underground laboratory (LSC, Laboratorio Subterràneo de Canfranc, Spain)^28^. Generally speaking, extension at the tidal frequencies includes the response of the solid Earth to the forcing tidal potential (solid tides) and to the water load oscillations caused by ocean tides (ocean loading), their sum giving tidal strain, as well as the response to environmental parameter changes (e. g., temperature and pressure) and other processes (e.g., hydrological processes like rain and snow melting). The interferometers measure local (tens-of-meter scale) strain directly, but local strain is biased by siting effects (e. g., cavity effects due to tunnel installation, surface topography, and rock inhomogeneities)^29,30^. Coupling between local extension *ε* (measured by the interferometers) and regional deformation *ε*_*ij*_ can be effectively described by three coefficients *α*, *β*, and *γ* per interferometer so that

2 $$\varepsilon =\alpha \,{\varepsilon }_{xx}+\beta \,{\varepsilon }_{yy}+\gamma \,{\varepsilon }_{xy}$$

where the *x*-axis is directed along the interferometer, the *y*-axis is perpendicular to that, and *ε*_*xy*_ is shear strain^31^.

Extension along any azimuthal direction *η* is:

3 $$\varepsilon (\eta )=\frac{{\varepsilon }_{\theta \theta }+{\varepsilon }_{\varphi \varphi }}{2}+\frac{{\varepsilon }_{\theta \theta }-{\varepsilon }_{\varphi \varphi }}{2}\,\cos \,\mathrm{(2}\eta )-{\varepsilon }_{\theta \varphi }\,\sin \,\mathrm{(2}\eta \mathrm{)}.$$

where *ε*_*θθ*_, *ε*_*ϕϕ*_, *ε*_*θϕ*_ are the horizontal components of the strain tensor in spherical coordinates (*r*, *θ*, *ϕ*), unit vectors $\hat{\theta }$ and $\hat{\varphi }$ being directed southward and eastward respectively. Complex amplitudes of diurnal strain solid tides (phase being given relative to the local tidal potential) are (see Data and Methods):

4 $$\begin{array}{ccl}\frac{{\varepsilon }_{\theta \theta }+{\varepsilon }_{\varphi \varphi }}{2} & = & 3\sqrt{\frac{5}{24\pi }}\frac{|{H}_{f}|}{a}A{\rm{s}}{\rm{i}}{\rm{n}}\theta {\rm{c}}{\rm{o}}{\rm{s}}\theta \\ \frac{{\varepsilon }_{\theta \theta }-{\varepsilon }_{\varphi \varphi }}{2} & = & -3\sqrt{\frac{5}{24\pi }}\frac{|{H}_{f}|}{a}(S+C)\sin \,\theta cos\theta \\ {\varepsilon }_{\theta \varphi } & = & -3\,i\sqrt{\frac{5}{24\pi }}\frac{|{H}_{f}|}{a}(S-C)\sin \,\theta ,\end{array}$$

where ${H}_{f}$ is the amplitude of the tidal term of frequency $f$ and $A$ is the mean Earth radius.

Complex parameters $A$, $S$, and $C$ (*A* being related to areal strain, $S$ and $C$ being related to the two shear strain components) are expressed in terms of the latitude-dependent Love and Shida numbers and follow the same resonance formula (Eq. 1), thus:

$$A(\sigma )={A}_{0}+\mathop{\sum }\limits_{j\mathrm{=1}}^{3}\frac{{A}_{j}}{(\sigma -{\sigma }_{j})},$$

and analogously for *S* and *C*, where *j* = 2 gives the effect of FCN. CW effects are almost constant and FICN effects are very small in the diurnal tidal band (e.g.^3^); thus, we neglect FICN effects, regard CW ones as constant ($\sigma -{\sigma }_{1}\approx 1-{\sigma }_{1}$) and focus on the FCN contribution to *A*, *S* and *C*, i.e. *A*_2_, *S*_2_ and *C*_2_.

The experimental data set consists of amplitudes and phases (i.e., complex amplitudes) of Q_1_, O_1_, P_1_, K_1_, ${\Psi }_{1}$, ${\Phi }_{1}$, N_2_ and M_2_ at each interferometer, as retrieved from the tidal analysis of strain records. Although the semidiurnal tides N_2_ and M_2_ are not affected by the FCN resonance, they have been considered to help evaluating siting effects on deformation. Optimal model parameters are obtained through the comparison between the experimental data set and theoretical diurnal tides (Eqs. 3 and 4) corrected for ocean loading and siting effects (Eq. 2). The comparison is quantified both through the sum of the magnitudes of the differences between experimental and theoretical tides (*L*^1^-norm) and through the sum of their squared magnitudes (*L*^2^-norm). Optimization through the *L*^2^-norm is more usual (e.g.^11^) but also much more affected by outliers than optimization through the *L*^1^-norm (e.g.^24^). Because of that, as a drawback, the use of *L*^1^-norm generally produces wider probability density functions (PDFs) of the inverted model parameters. We estimate PDFs and possible correlations of inverted parameters following a frequentist approach based on the inversion of thousands of synthetic data sets which we obtain from the experimental one by adding a gaussian random noise whose standard deviation is given by the tidal analysis of the first-difference strain sequences (see Data and Methods).

In what follows we use the expression “reference value” to indicate the value of each parameter if computed by using reference IERS^3^ frequency-dependent Love and Shida numbers at 42.6° latitude, i. e. between LNGS and LSC (Fig. 1a).

## Results

In principle, we would like to retrieve the following model parameters:

- *T*_*FCN*_ and *Q*_*FCN*_;
- ${A}_{0}+{A}_{1}\mathrm{/(1}-{\sigma }_{1})$, ${S}_{0}+{S}_{1}\mathrm{/(1}-{\sigma }_{1})$, ${C}_{0}+{C}_{1}\mathrm{/(1}-{\sigma }_{1})$, ${A}_{2}$, ${S}_{2}$, and *C*_2_ at each station;
- *α*, *β*, and *γ* for each interferometer.

**Figure 2:**
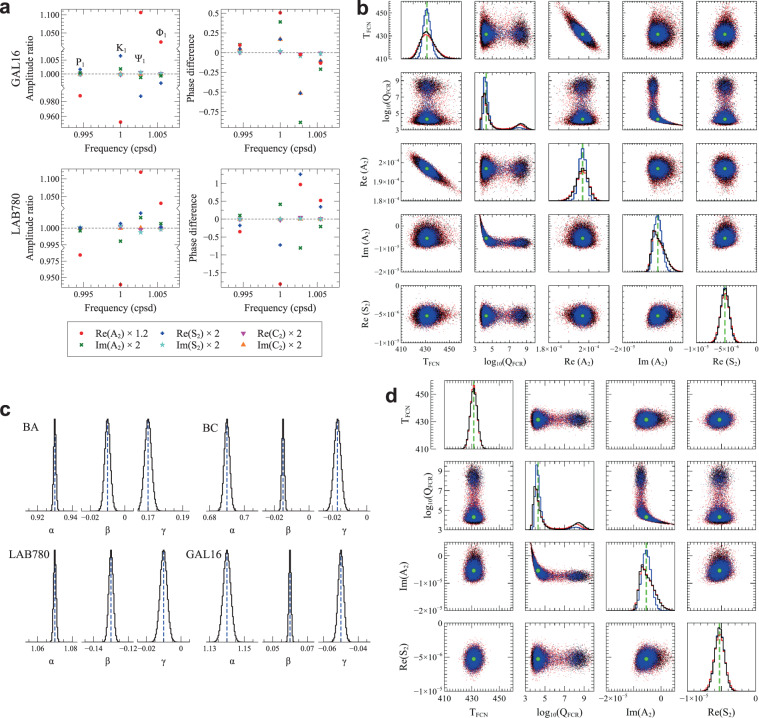
Resolving power of tidal strain data. (**a**) Amplitude ratio and phase difference between solid tides computed along LAB780 and GAL16 using reference ${A}_{2}$, ${S}_{2}$, ${C}_{2}$ values and after increasing one by one ${\rm{Re}}({A}_{2})$ by 20% and ${\rm{Im}}({A}_{2})$, ${\rm{Re}}({S}_{2})$, ${\rm{Im}}({S}_{2})$, ${\rm{Re}}({C}_{2})$, ${\rm{Im}}({C}_{2})$ by 100%. Results for BA and BC are quite similar because BA is almost parallel to LAB780 and BC to GAL16. (**b**,**c**) Results of the validation of the inversion procedure when parameters ${T}_{FCN}$, ${\log }_{10}({Q}_{FCN})$, ${\rm{Re}}({A}_{2})$, ${\rm{Im}}({A}_{2})$, and ${\rm{Re}}({S}_{2})$, as well as siting coefficients at each interferometer, are left free (see Data and Methods). Black (red) lines and dots refer to ${L}^{2}$-norm (${L}^{1}$-norm) inversions; blue lines and dots refer to ${L}^{2}$-norm results obtained after halving ${\Psi }_{1}$ and ${\Phi }_{1}$ uncertainties. Main diagonal plots in panel b show PDFs; off-diagonal scatter plots (limited to 10000 points for clarity) show correlations between parameters; the panel also includes reference parameter values, as green dashed lines in the main diagonal plots and green circles in the off-diagonal scatter plots. Plots in panel c give the PDFs of the 12 siting coefficients with arbitrary y-axis scale; blue dashed lines give the values used to generate the inverted data set. (**d**) Results from the inversion of the same data set as before, but fixing ${\rm{Re}}({A}_{2})$ to its reference value.

Unfortunately, such a goal is practically unreachable, not only because of the large number of parameters. We assume that *A*_*j*_, *S*_*j*_, and *C*_*j*_ are the same for both stations since the latitudes of LNGS and LSC are very close to each other (Fig. 1a). Moreover, it is intrinsically impossible to retrieve ${A}_{0}+{A}_{1}\mathrm{/(1}-{\sigma }_{1})$, ${S}_{0}+{S}_{1}\mathrm{/(1}-{\sigma }_{1})$, and ${C}_{0}+{C}_{1}\mathrm{/(1}-{\sigma }_{1})$, as well as *α*, *β*, and *γ* from strain tides simultaneously; thus, we fix ${A}_{0}+{A}_{1}\mathrm{/(1}-{\sigma }_{1})$, ${S}_{0}+{S}_{1}\mathrm{/(1}-{\sigma }_{1})$, and ${C}_{0}+{C}_{1}\mathrm{/(1}-{\sigma }_{1})$ to their reference values. A study of tidal strain sensitivity to *A*_2_, *S*_2_, *C*_2_ indicates that the effects of Im(*S*_2_), Re(*C*_2_), and Im(*C*_2_) are too small to be detectable and tidal strain is mainly sensitive to ${\rm{Re}}({A}_{2})$ (Fig. 2a).

Firstly we carry out validation tests on a data set constituted by theoretical body tides to which we attribute the same uncertainties as the experimental tides, showing that the inversion technique produces reliable estimates of *T*_*FCN*_, *Q*_*FCN*_ (actually, log_10_ (*Q*_*FCN*_)), Re(*A*_2_), ${\rm{Im}}({A}_{2})$, ${\rm{Re}}({S}_{2})$, and, for each interferometer, *α*, *β*, and *γ* (see Data and Methods and Fig. 2b,c). However, there is a strong trade-off (linear correlation) between Re(*A*_2_) and *T*_*FCN*_, with a *T*_*FCN*_ increase by 10 sidereal days when Re(*A*_2_) decreases by about 5%. A similar behaviour is also observed when inverting the experimental data set; thus, it is difficult to estimate both Re(*A*_2_) and *T*_*FCN*_ without strong a priori constraints. Since PDF of Re(*A*_2_) from the experimental data set agrees with its reference value, regardless of the ocean and Earth models used to compute ocean loading (see Data and Methods), we choose to fix Re(*A*_2_) in the final inversions; such a choice does not affect PDFs and correlations related to the other parameters (Fig. 2d). No other trade-off related to *T*_*FCN*_ is visible. The trade-off between *Q*_*FCN*_ and ${\rm{Im}}({A}_{2})$ is limited to small values of *Q*_*FCN*_ (*Q*_*FCN*_ < 10^4^), thus we leave ${\rm{Im}}({A}_{2})$ as a free parameter in the inversion. PDF of ${\log }_{10}({Q}_{FCN})$ is bimodal, exhibiting a much smaller secondary mode at large *Q*_*FCN*_ values. This secondary mode is related to synthetic data sets where complex amplitudes of ${\Psi }_{1}$ and ${\Phi }_{1}$ are very different from the starting (theoretical) ones; it lowers noticeably when uncertainties (and consequently widths of the random noise used to generate synthetic data sets) on ${\Psi }_{1}$ and ${\Phi }_{1}$ are halved. Moreover, as discussed in^24^, the only appreciable effect of decreasing *Q*_*FCN*_ from very large values down to 20,000 is a change of the ${\Psi }_{1}$ phase by less than 2°, i. e., much less than its uncertainty. As a consequence, median of *Q*_*FCN*_ from the inversion of synthetic data sets (19,943) practically coincides with the value used to generate the theoretical data set (20,000), but the 95% confidence interval is 5,224 to 6 × 10^8^. When Re(*A*_2_) is fixed to its reference value in the inversions, PDFs and correlations involving *T*_*FCN*_ are narrower than when Re(*A*_2_) is left free; all the other PDFs and correlations remain nearly unchanged (see Fig. 2d).

Secondly we invert the experimental data set, but, before showing related results, a couple of points have to be discussed.

**Figure 3:**
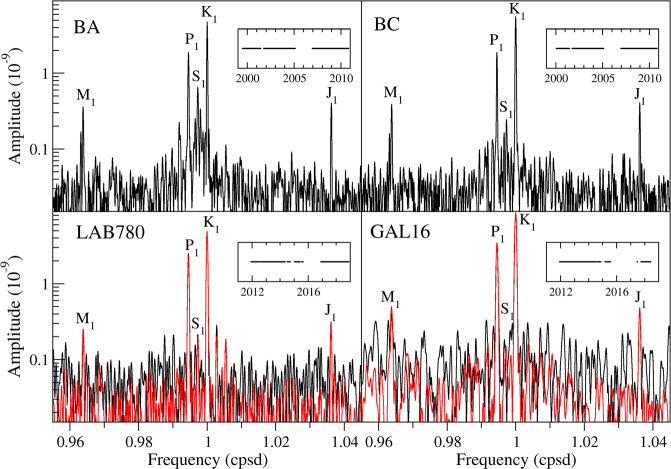
Strain amplitude spectrum^42^ inside the diurnal tidal band for each interferometer. Insets show the analysed time intervals. As for LAB780 and GAL16, red lines give spectra after removing time periods when the deformation signal is affected by snow melting and heavy rain episodes (“cleaned” data). Frequencies are expressed in cycles per sidereal day. Main diurnal tidal constituents are labelled.

(i) In principle, changes in environmental and hydrological parameters could affect our results. As for the LNGS interferometers (BA and BC), we already performed several attempts to correct strain for barometric pressure and air temperature, by comparing spectra of residuals from the joint tidal analysis of strain and air temperature or barometric pressure with those from the sole strain^24^. Noise level did not lower and we did not correct strain records for temperature and pressure fluctuations; indeed, the BA peak around S_1_ (Fig. 3) is probably of anthropic origin. As for the LSC interferometers (LAB780 and GAL16), we already demonstrated that air temperature and barometric pressure effects on strain are actually negligible on time scales of one day or shorter^28,32^. However, strain records show clear signatures of late-spring snow melting, characterized by daily deformation cycles lasting few weeks, and heavy rain episodes, characterized by several-hour-long deformation pulses (manuscript in preparation, but see^33^ for a preliminary study). Removing the affected time periods from both LAB780 and GAL16 data approximately halves the noise amplitude in the diurnal band (red lines in Fig. 3), but decreases the number of analysed data by almost 10% and introduces several additional breaks into the strain sequences; as a consequence, it is difficult to foresee whether results improve or do not. Those cut strain records are hereinafter referred to as “cleaned” data.

(ii) Ocean loading mainly affects semidiurnal tides at LSC (Fig. 1b), while giving a small even though not negligible contribution to the other tides. Although computed ocean loading depends on the ocean and Earth models that are used in the computation (see Data and Methods), results obtained with tested models are always within few percent in amplitude and few degrees in phase. These small differences affects inversion misfits (Fig. 1c) but have a small influence on PDFs of model parameters (see later in the text). In what follows, ocean loading is computed using the combination of ocean and Earth models that gives the smallest misfit (i. e., FES2014b^34^ and continental PREM^35,36^) unless otherwise specified.

**Figure 4:**
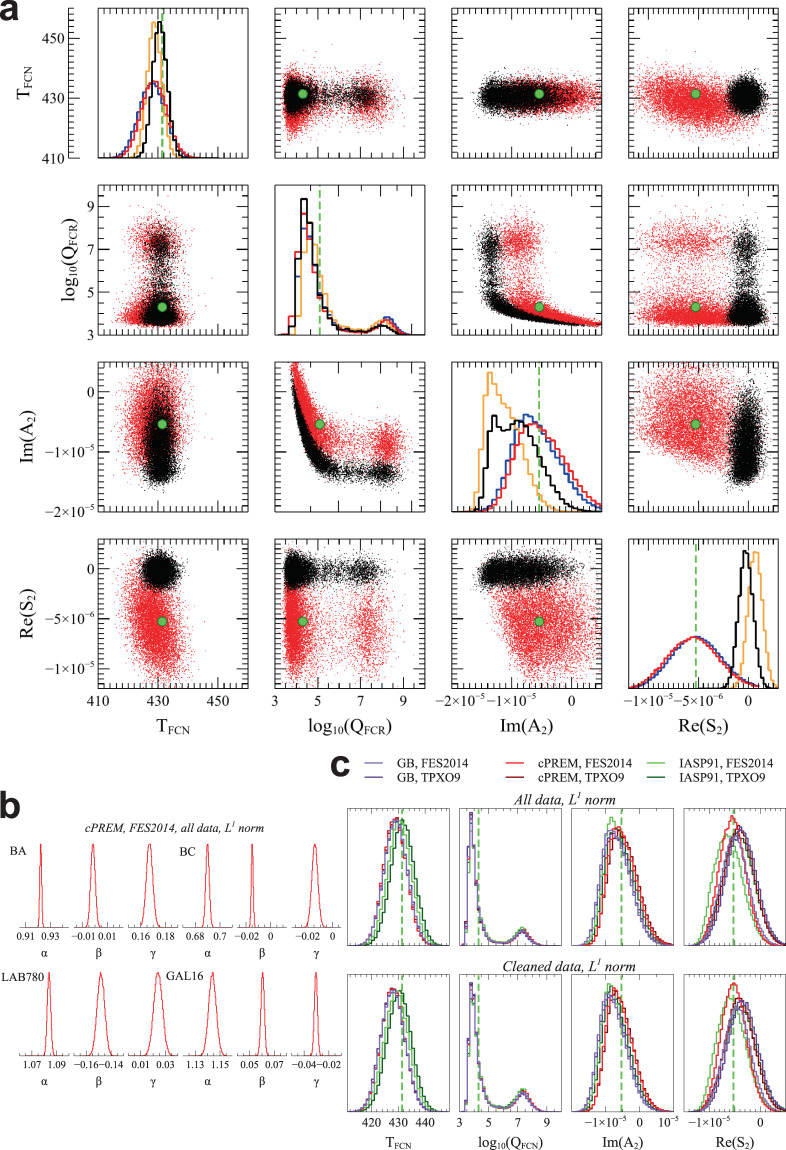
Model parameters and siting coefficients from the inversion of the experimental tidal set. (**a**) Main diagonal plots show PDFs with arbitrary y-axis scale; off-diagonal scatter plots (limited to 10000 points for clarity) show correlations between parameters; the panel also includes reference parameter values, as green dashed lines in the main diagonal plots and green circles in the off-diagonal scatter plots. Red (black) lines and dots refer to ${L}^{1}$-norm (${L}^{2}$-norm) inversions of tides obtained from the analysis of all strain data; blue (orange) lines refer to ${L}^{1}$-norm (${L}^{2}$-norm) inversions of tides obtained from the analysis of “cleaned” strain data. Ocean loading is computed using FES2014 ocean model and cPREM Earth model. (**b**) PDFs of the 12 siting coefficients with arbitrary y-axis scale. (**c**) PDFs of model parameters (arbitrary y-axis scale) for different Earth and ocean models (see Data and Methods), ${L}^{1}$-norm inversions of both all and “cleaned” strain data.

Figure 4a shows PDFs and correlations of the free model parameters when we invert the experimental data set obtained from the analysis of all strain data. All ${L}^{1}$-norm PDFs and correlations are fully consistent with, although wider than, those obtained when inverting the data set based on theoretical body tides (Fig. 2d); PDF of ${\log }_{10}({Q}_{FCN})$ is somewhat shifted toward smaller values. Siting coefficients are well constrained (Fig. 4b); for the first time ${K}_{1}$ (the largest diurnal strain tide) is also used to estimate them: since ${K}_{1}$ is heavily influenced by the FCN, we could do that just because the siting coefficient estimate is included into the tidal parameter inversion procedure. The ${L}^{2}$-norm PDFs of ${\rm{Re}}({S}_{2})$ and ${\rm{Im}}({A}_{2})$ are shifted toward positive and negative values respectively, probably as a consequence of the strong sensitivity of the ${L}^{2}$-norm inversion to the presence of outliers. This strong difference between ${L}^{1}$- and ${L}^{2}$-norm results does not occur in the validation tests, where synthetic data sets used for the PDFs are obtained by adding a gaussian noise to “exact” tidal complex amplitudes.

**Figure 5:**
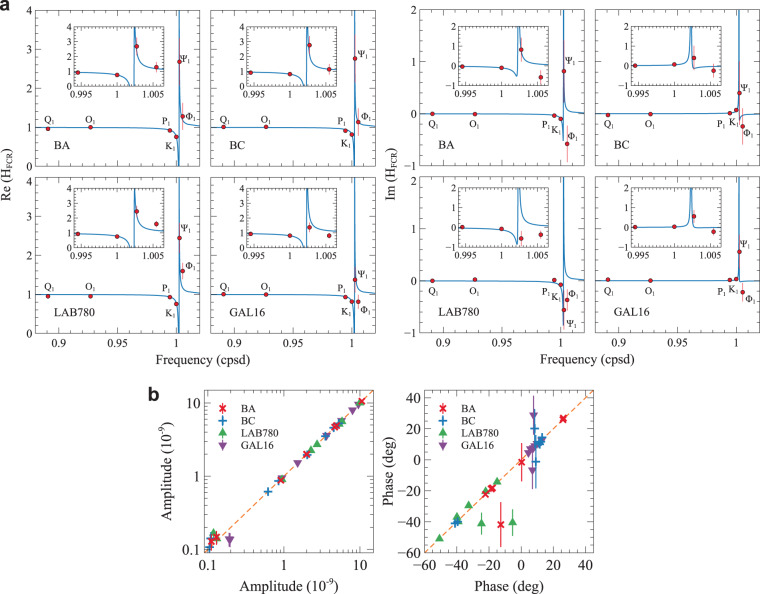
Comparison between observations and theory. (**a**) Real and imaginary parts of the observed (red dots) and theoretical (blue lines) complex transfer function H_*FCN*_. Error bars (twice the standard deviations) are shown. (**b**) Correlation plots between theoretical and experimental amplitudes and phases (with respect to local tidal potential) of Q_1_, O_1_, P_1_, K_1_, ${\Psi }_{1}$, ${\Phi }_{1}$, N_2_ and M_2_, for each interferometer. Experimental amplitudes and phases are from the tidal analysis of all strain data.

To see how measured tides agree with modelled tides as computed using the median values of the ${L}^{1}$-norm parameter PDFs (Fig. 4a), we compare the observed and theoretical complex transfer functions (${{\rm{H}}}_{FCN}$) of the FCN for each interferometer (Fig. 5a). Observed ${{\rm{H}}}_{FCN}$ is the ratio of observed solid tides (i. e., observed tides corrected for ocean loading) on computed solid tides without resonance, i. e., putting ${A}_{2}={S}_{2}={C}_{2}\mathrm{=0}$ in Eq. 4. Theoretical ${{\rm{H}}}_{FCN}$ is the ratio of modelled solid tides (computed using the median values of the FCN parameter PDFs from the ${L}^{1}$-norm inversion of tides obtained from the analysis of all strain data and corrected for siting effects) on computed solid tides without resonance. For both observed and theoretical ${{\rm{H}}}_{FCN}$, solid tides without resonance are corrected for siting effects (Eq. 2) using the medians of the PDFs of $\alpha$, $\beta$, $\gamma$ at each interferometer (Fig. 4b). The fit is very good for all the tidal harmonics, except ${\Psi }_{1}$ and ${\Phi }_{1}$, at all the interferometers. However, taking into account the large uncertainties in the ${\Psi }_{1}$ and ${\Phi }_{1}$ ocean loading effects, which are not included in the error bars and might bias theoretical tidal strain, the agreement is still reasonable for all the interferometers but ${\rm{Im}}({{\rm{H}}}_{FCN})$ for LAB780. The FCN effect on ${K}_{1}$ strain is large (up to 25% in amplitude and 7° in phase, depending on the interferometer) thus robustness of retrieved model parameters is also grounded on the major ${K}_{1}$ tide.

Correlation plots between theoretical and experimental tides confirm the aforementioned results (Fig. 5b). In this comparison, theoretical tides are computed using the medians of the FCN parameter PDFs (${L}^{1}$-norm inversion of tidal complex amplitudes obtained from the analysis of all strain data, Fig. 4a), corrected for ocean loading and siting effects. Again, siting effects are computed using the medians of the PDFs of $\alpha$, $\beta$, $\gamma$ at each interferometer (Fig. 4b).

Using different ocean and Earth models or analysing “cleaned“ data produces practically identical ${L}^{1}$-norm PDFs (Fig. 4c); siting coefficients vary by no more than few 10^−2^. We obtain final ${T}_{FCN}$ and ${\log }_{10}({Q}_{FCN})$ PDFs by stacking all the histograms in Fig. 4c; medians and 95% confidence intervals are listed in Table 1.

## Discussion and Conclusions

We have inverted the complex amplitudes of six diurnal (Q_1_, O_1_, P_1_, K_1_, ${\Psi }_{1}$, ${\Phi }_{1}$) and two semidiurnal (N_2_, M_2_) strain tides per interferometer by using both ${L}^{1}$-norm and ${L}^{2}$-norm optimizations. The ${L}^{1}$-norm optimization gives almost indistinguishable results when we obtain the experimental data set from all strain data or after removing the time periods affected by hydrological processes from LSC records (“cleaned” data), as well as using different ocean (FES214 and TPXO9) and Earth (cPREM, GB, IASP91) models (Fig. 4c). Results obtained using the ${L}^{2}$-norm optimization are less stable. This behaviour is not surprising because non-stochastic ill-modelled processes (e.g. ocean loading, hydrologically-induced deformation, changes in air temperature and pressure) can contribute to the mismatch between theoretical and measured tides, thus affecting ${L}^{2}$-norm optimization to a larger extent than ${L}^{1}$-norm optimization. Figure 4a,c indicate that using the ${L}^{1}$-norm effectively reduces the impact of poorly known contributions to the tidal signal on inversion results. Because of that, we consider ${L}^{1}$-norm results more reliable than ${L}^{2}$-norm ones, although ${L}^{1}$-norm PDFs are wider.

Amplitudes of ${\Psi }_{1}$ and ${\Phi }_{1}$ are very small, thus their relative uncertainties on amplitudes and absolute uncertainties on phases are much larger than for the other tides involved in the inversion. If uncertainties on ${\Psi }_{1}$ and ${\Phi }_{1}$ are halved when we invert the data set constituted by theoretical body tides (blue lines and dots in Fig. 2b), then correlation between ${\rm{Re}}({A}_{2})$ and ${T}_{FCN}$ decreases, PDFs of ${T}_{FCN}$, ${\rm{Re}}({A}_{2})$ and ${\rm{Im}}({A}_{2})$ become almost symmetrical, and the secondary mode of ${\log }_{10}({Q}_{FCN})$ PDF lowers noticeably; PDF of ${\rm{Re}}({S}_{2})$ does not change appreciably. Medians of all PDFs also do not change appreciably and are quite similar to the reference values used to compute the theoretical body tides. It is noteworthy that halving ${\Psi }_{1}$ and ${\Phi }_{1}$ uncertainties has no visible effect on the PDF of ${T}_{FCN}$ when ${\rm{Re}}({A}_{2})$ is fixed (Fig. 2d). Because of the monochromatic nature of tides, halving uncertainties on experimental ${\Psi }_{1}$ and ${\Phi }_{1}$ would require analysing strain records four times as long. PDF of ${T}_{FCN}$ in this work is about half as wide as in^24^ mainly because of the inclusion of LSC data in the analysis, while the use of more recent ocean and Earth models and improvements in the inversion procedure have produced no significant change in the ${T}_{FCN}$ median.

As previously mentioned, gravity data have been extensively used to investigate FCN through its resonance effects on tides. Strain tidal measurements have a lower signal-to-noise ratio than gravity, but relative perturbations on strain tides are about 10 times larger than on gravity tides and inversions of synthetic data sets suggest that the resolving power of gravity and strain tides are comparable^24^. There is a correlation between ${T}_{FCN}$ and the real part of the resonance strength in gravity tides ${\rm{Re}}({\delta }_{2})$^10,11^: since ${\rm{Re}}({\delta }_{2})$ strongly depends on ${h}_{2}$^2^, this correlation is consistent with that between ${T}_{FCN}$ and ${\rm{Re}}({A}_{2})$ in strain tides (Fig. 2b). Moreover, ${\rm{Im}}({\delta }_{2})$ is correlated with ${Q}_{FCN}$ at small values of ${Q}_{FCN}$^10^, like ${\rm{Im}}({A}_{2})$ is.

We are conscious that there are some (currently) unavoidable inconsistencies in our approach. As for theoretical solid tides, they are computed using Love and Shida numbers based on a Spherical Non Rotating Elastic Isotropic (SNREI) Earth model (PREM) and modified to account for ellipticity and rotation^3^. Computations of solid tides using a full 3D Earth model with mantle heterogeneities evidence differences <0.1% in gravity and vertical displacement with respect to a radially symmetric Earth model^37^: these differences are small and eventually counterbalanced by siting effect corrections, which are estimated during the inversion procedure. However, because of the trade-off between ${\rm{Re}}({A}_{2})$ and ${T}_{FCN}$, ${\rm{Re}}({A}_{2})$ is fixed to its reference value from parameters in^3^, which were obtained from VLBI data using geophysical models available at the end of the 20th century, and any change in ${\rm{Re}}({A}_{2})$ would imply a change in ${T}_{FCN}$. As for ocean loading, strain Green functions from SNREI Earth models (GB, cPREM, IASP91) are used. A more correct approach would involve computing deformation using a full 3D Earth model, which should be particularly accurate at short distances from the stations. This is an important point, even though Fig. 4c suggests that PDFs of ${T}_{FCN}$ and ${\log }_{10}({Q}_{FCN})$ are robust, presumably because of the small contribution of ocean loading to diurnal tides at LSC and LNGS.

In conclusion, data analysed in this work (strain tides) are quite different from those (forced nutations and gravity tides) generally used to estimate FCN parameters (period, quality factor, resonance strengths) and have a different sensitivity to some of them. The inversion approach is also different, being based on robust ${L}^{1}$-norm misfit minimization. All the ${T}_{FCN}$ PDFs, which we obtain using different ocean and Earth models to compute the ocean loading contribution to tides, agree with reference values^3^ and estimates from previous analyses of nutations and gravity (Table 1). Median values from GB and cPREM Earth models are somewhat smaller than reference ${T}_{FCN}$, but IASP91 results are in full agreement with most of recent estimates from nutation. Although the final PDF is wider than those from the analyses of nutations, this work provides an alternative independent estimate of ${T}_{FCN}$. Accurate estimates of ${Q}_{FCN}$ come from nutations only, nevertheless medians of the distributions in Fig. 4c are close to (actually, somewhat smaller than) its reference value^3^. Parameters ${\rm{Im}}({A}_{2})$ and ${\rm{Re}}({S}_{2})$ are estimated in this work for the first time; their PDFs are in agreement with reference values computed using Love and Shida numbers from^3^.

## Data and Methods

### Tidal displacements and strain

Here we give the diurnal and semidiurnal degree-2 displacement tides for an elliptical rotating Earth with an imperfectly elastic mantle as in^3^, Equations 7.1b and 7.1c. Differently from^3^, we use spherical coordinates ($r$, $\theta$, $\varphi$), where the unit vectors $\hat{\theta }$ and $\hat{\varphi }$ are directed southward and eastward respectively. We get the three components of the surface strain tensor (${\varepsilon }_{\theta \theta }$, ${\varepsilon }_{\varphi \varphi }$ and ${\varepsilon }_{\theta \varphi }$, Eq. 4) in spherical coordinates after taking the spatial derivatives of displacements; the Earth surface is approximated as spherical (radius *a*) in the derivatives.

Because of the Earth’s rotation and flattening, spheroidal displacements of degree 2 order 1 (diurnal degree-2 tides) are coupled to spheroidal displacements of degree 4 and toroidal displacements of degree 1 and 3. If diurnal tidal displacements are given with respect to the potential evaluated on a spherical surface, spheroidal displacements are expressed in terms of latitude-dependent Love ($h(\theta )$) and Shida ($l(\theta )$) numbers, and toroidal displacements are expressed in terms of the additional numbers ${l}^{\mathrm{(1)}}$ and *l*′, this latter acting as a no-net-Earth-rotation correction^38^.

For a diurnal tide of frequency $f$:

$$\begin{array}{ccl}\Delta {\overrightarrow{r}}_{f} & = & \sqrt{\frac{5}{96\pi }}{H}_{f}\,\{3h(\theta ){\sin }^{2}\theta \,\cos ({\theta }_{f}+2\varphi )\,\hat{r}\\  &  & +\,6\,\sin \,\theta \,\cos \,\theta [l(\theta )+\,{l}^{(1)}]\,\cos ({\theta }_{f}+2\varphi )\,\hat{\theta }\\  &  & -\,6\,\sin \,\theta [l(\theta )+{l}^{(1)}{\cos }^{2}\theta ]\,\sin ({\theta }_{f}+2\varphi )\hat{\varphi }\}.\end{array}$$

where:

$$h(\theta )={h}^{\mathrm{(0)}}+{h}^{\mathrm{(2)}}(3{\cos }^{2}\theta -1)\mathrm{/2,}$$

$$l(\theta )={l}^{\mathrm{(0)}}+{l}^{\mathrm{(2)}}(3{\cos }^{2}\theta -1)\mathrm{/2,}$$

$${H}_{f}={\rm{amplitude}}\,({\rm{m}})\,{\rm{of}}\,{\rm{the}}\,{\rm{tidal}}\,{\rm{term}}\,{\rm{of}}\,{\rm{frequency}}\,f,$$

$$\theta ={\rm{g}}{\rm{e}}{\rm{o}}{\rm{c}}{\rm{e}}{\rm{n}}{\rm{t}}{\rm{r}}{\rm{i}}{\rm{c}}\,{\rm{c}}{\rm{o}}{\rm{l}}{\rm{a}}{\rm{t}}{\rm{i}}{\rm{t}}{\rm{u}}{\rm{d}}{\rm{e}}\,{\rm{o}}{\rm{f}}\,{\rm{s}}{\rm{t}}{\rm{a}}{\rm{t}}{\rm{i}}{\rm{o}}{\rm{n}},$$

$$\varphi ={\rm{east}}\,{\rm{longitude}}\,{\rm{of}}\,{\rm{station}},$$

$${\theta }_{f}={\rm{t}}{\rm{i}}{\rm{d}}{\rm{e}}\,{\rm{a}}{\rm{r}}{\rm{g}}{\rm{u}}{\rm{m}}{\rm{e}}{\rm{n}}{\rm{t}}\,{\rm{f}}{\rm{o}}{\rm{r}}\,{\rm{t}}{\rm{i}}{\rm{d}}{\rm{a}}{\rm{l}}\,{\rm{c}}{\rm{o}}{\rm{n}}{\rm{s}}{\rm{t}}{\rm{i}}{\rm{t}}{\rm{u}}{\rm{e}}{\rm{n}}{\rm{t}}\,{\rm{w}}{\rm{i}}{\rm{t}}{\rm{h}}\,{\rm{f}}{\rm{r}}{\rm{e}}{\rm{q}}{\rm{u}}{\rm{e}}{\rm{n}}{\rm{c}}{\rm{y}}\,f,$$

$$\hat{r}={\rm{unit}}\,{\rm{vector}}\,{\rm{in}}\,{\rm{radial}}\,{\rm{direction}},$$

$$\hat{\theta }={\rm{u}}{\rm{n}}{\rm{i}}{\rm{t}}\,{\rm{v}}{\rm{e}}{\rm{c}}{\rm{t}}{\rm{o}}{\rm{r}}\,{\rm{p}}{\rm{e}}{\rm{r}}{\rm{p}}{\rm{e}}{\rm{n}}{\rm{d}}{\rm{i}}{\rm{c}}{\rm{u}}{\rm{l}}{\rm{a}}{\rm{r}}\,{\rm{t}}{\rm{o}}\,\hat{r}\,{\rm{i}}{\rm{n}}\,{\rm{t}}{\rm{h}}{\rm{e}}\,{\rm{s}}{\rm{o}}{\rm{u}}{\rm{t}}{\rm{h}}{\rm{w}}{\rm{a}}{\rm{r}}{\rm{d}}\,{\rm{d}}{\rm{i}}{\rm{r}}{\rm{e}}{\rm{c}}{\rm{t}}{\rm{i}}{\rm{o}}{\rm{n}},$$

$$\hat{\varphi }={\rm{unit}}\,{\rm{vector}}\,{\rm{in}}\,{\rm{east}}\,{\rm{direction}}\mathrm{}.$$

After taking the spatial derivatives of displacements, we get Eq. 4. Parameters *A*, *S*, and *C* are:

$$\begin{array}{ccc}A & = & \left[{h}^{\mathrm{(0)}}+\frac{1}{2}(3{\cos }^{2}\theta -1){h}^{\mathrm{(2)}}\right]-3\left[{l}^{\mathrm{(0)}}+\left(\frac{5}{2}{\cos }^{2}\theta -1\right){l}^{\mathrm{(2)}}\right]-\frac{{l}^{\mathrm{(1)}}}{2},\\ S & = & {l}^{\mathrm{(0)}}+\frac{5}{4}(3{\cos }^{2}\theta -1){l}^{\mathrm{(2)}}+\frac{1}{2}({\cos }^{2}\theta +1){l}^{\mathrm{(1)}},\\ C & = & \frac{3}{4}({\cos }^{2}\theta -1){l}^{\mathrm{(2)}}-\frac{1}{2}({\cos }^{2}\theta -2){l}^{\mathrm{(1)}}\mathrm{}.\end{array}$$

Parameter *l*′ does not appear in the expressions for the strain because it refers to purely rotational displacements.

Parameters ${h}^{\mathrm{(0)}}$, ${h}^{\mathrm{(2)}}$, ${l}^{\mathrm{(0)}}$, ${l}^{\mathrm{(1)}}$, ${l}^{\mathrm{(2)}}$, and $l{\prime}$ follow the resonance formula given in Eq. 1; numerical values based on the preliminary reference Earth model (PREM^35^) corrected for mantle anelasticity^39^ are in^3^. FCN effects on $A$, $S$ and $C$ are represented by ${A}_{2}$, ${S}_{2}$ and ${C}_{2}$: from Table 7.1 in^3^, we see that the main contribution to the (complex) ${A}_{2}$ term comes from ${h}_{2}^{\mathrm{(0)}}$, the main contribution to the (complex) ${S}_{2}$ term comes from ${l}_{2}^{\mathrm{(0)}}$, and the main contribution to the (complex) ${C}_{2}$ term comes from ${l}_{2}^{\mathrm{(1)}}$.

We also give expressions for semidiurnal tides because we use them to evaluate siting effects on deformation. Spheroidal displacements of degree 2 order 2 (semidiurnal degree-2 tides) are coupled to spheroidal displacements of degree 4 and toroidal displacements of degree 3.

For a semidiurnal tide of frequency *f*:

$$\begin{array}{ccc}\Delta {\overrightarrow{r}}_{f} & = & \sqrt{\frac{5}{96\pi }}{H}_{f}\,\{3h(\theta ){\sin }^{2}\theta \,\cos ({\theta }_{f}+2\varphi )\,\hat{r}\\  &  & +6\,\sin \,\theta \,\cos \,\theta [l(\theta )+{l}^{(1)}]\,\cos ({\theta }_{f}+2\varphi )\,\hat{\theta }\\  &  & -6\,\sin \,\theta [l(\theta )+{l}^{(1)}{\cos }^{2}\theta ]\,\sin ({\theta }_{f}+2\varphi )\hat{\varphi }\}.\end{array}$$

After taking the spatial derivatives of semidiurnal displacements, we get

5 $$\begin{array}{ccc}\frac{{\varepsilon }_{\theta \theta }+{\varepsilon }_{\varphi \varphi }}{2} & = & \frac{3}{2}\sqrt{\frac{5}{24\pi }}\frac{|{H}_{f}|}{a}\left[{h}^{\mathrm{(0)}}+\frac{(3{\cos }^{2}\theta -1)}{2}{h}^{\mathrm{(2)}}-3{l}^{\mathrm{(0)}}-{l}^{\mathrm{(1)}}-\frac{3}{2}(4-5{\sin }^{2}\theta ){l}^{\mathrm{(2)}}\right]{\sin }^{2}\theta \\ \frac{{\varepsilon }_{\theta \theta }-{\varepsilon }_{\varphi \varphi }}{2} & = & \frac{3}{2}\sqrt{\frac{5}{24\pi }}\frac{|{H}_{f}|}{a}\left[(2-{\sin }^{2}\theta ){l}^{\mathrm{(0)}}+(2-3{\sin }^{2}\theta ){l}^{\mathrm{(1)}}+\left(2-7{\sin }^{2}\theta +\frac{9}{2}{\sin }^{4}\theta \right){l}^{\mathrm{(2)}}\right]\\ {\varepsilon }_{\theta \varphi } & = & 3\,i\,\sqrt{\frac{5}{24\pi }}\frac{|{H}_{f}|}{a}[{l}^{\mathrm{(0)}}+(1-3{\sin }^{2}\theta ){l}^{\mathrm{(2)}}+{\cos }^{2}\theta \,{l}^{\mathrm{(1)}}]\cos \,\theta \end{array}$$

### Strain data

We use strain records from two pairs of high-sensitivity laser strainmeters (interferometers, Fig. 1a). The older pair (named BA and BC) has operated from summer 1999 to March 2013 under the Gran Sasso massif, Italy, inside the Gran Sasso underground laboratories (LNGS, Laboratori Nazionali del Gran Sasso)^27^. The two interferometers were developed on the basis of a previous instrument working in the same place from 1994 to 1999^40^, with some electronic and optical improvements; each interferometer was about 90 m long. The newer pair (named LAB780 and GAL16) is operating since winter 2011 under the Central Pyrenees, Spain, close to the Canfranc underground laboratory (LSC, Laboratorio Subterràneo de Canfranc)^28^. These two interferometers include further improvements in the mechanical and optical set-up with respect to the LNGS ones; each interferometer is about 70 m long. The LNGS and LSC interferometers are located at about the same latitude (42.46°and 42.76°, respectively) and have similar azimuthal directions (BA, −24^°^; BC, 66°; LAB780, −30.9°; GAL16, 77.4°), BA being about parallel to LAB780 and BC being about parallel to GAL16. Both pairs of instruments are based on the classical unequal-arm Michelson set-up and compare the optical length of a longer measurement arm to the one of a shorter fixed reference arm. They are characterized by very high sensitivity (nominal sensitivity $\Delta l/l$ of a few 10^−13^), wide frequency band (hundreds of Hz to continuum), and large dynamic range (more than 7 orders of magnitude), limited only by the capability of maintaining optical alignment.

Unfortunately, strain data acquisition suffered several interruptions at both sites because of technical problems, mainly due to laser tube replacements. Thus, we could analyse about 7 years of LNGS data from 2000 to 2011 and about 4.6 years of LSC data from 2012 to 2018 (see insets in Fig. 3).

### Strain record analysis

At first, we low-pass filter and decimate (1 sample per 1800 s) strain data from each interferometer, thus generating evenly spaced time series with gaps. Then, we pre-whiten each ungapped data segment by generating first-difference sequences, because deformation noise is red (i.e., its power spectral density increases as frequency decreases) while most tidal analysis techniques assume a gaussian residual distribution (i.e., a white noise spectrum) when fitting tidal harmonics and, e. g., polynomials to the data time series. Pre-whitening makes fitting more reliable as it lowers low-frequency noise, which is the most difficult to deal with having a statistically non-null mean value; first-difference strain sequences exhibit a noise spectrum which is almost white, e.g.^41^.

To estimate signal-to-noise (S/N) in the diurnal tidal band for each interferometer, we calculate the amplitude spectrum of the gapped first-difference time series through an iterative deconvolution of the spectral window in the frequency domain, by using CLEAN code^42^ (Fig. 3). Overall, spectra exhibit very small diurnal contamination, mainly as for LSC data, and very good S/N in the diurnal tidal band. Unfortunately, analogous detailed spectra are seldom published and thus it is difficult to make a direct comparison with other strainmeters.

We apply the tidal analysis code VAV version 5 (VAV05)^43^ to the first-difference time series to generate an experimental tidal parameter set, which consists of 10 diurnal and 6 semi-diurnal tidal groups, for each interferometer. The set includes amplitude and phase (with respect to the local potential) of Q_1_, O_1_, P_1_, K_1_, ${\Psi }_{1}$, ${\Phi }_{1}$, N_2_, M_2_, which are used as experimental tidal data set in the inversions. Tidal parameters are corrected for the effects of pre-whitening as follows:

$$\begin{array}{ccc}\phi  & = & {\phi }_{PW}-{90}^{\circ }\\ A & = & \frac{{A}_{PW}}{2\,\sin (2\pi f\Delta t/2)}\end{array}$$

where ${A}_{PW}$ (${\phi }_{PW}$) and *A* ($\phi$) are the amplitude (phase) for the pre-whitened and real series respectively, $f$ is frequency and $\varDelta t$ is sampling time^24^.

### Inversions, probability distributions and correlations of inverted parameters

We use 32 complex tidal amplitudes (Q_1_, O_1_, P_1_, K_1_, ${\Psi }_{1}$, ${\Phi }_{1}$, N_2_, M_2_ at each interferometer), thus the total number of data is 64; each amplitude and phase pair is converted into its cosine (real) and sine (imaginary) terms, so that their dependence on the local-effect coefficients is linear. The model parameter space is 16-dimensional (${T}_{FCN}$, ${log}_{10}({Q}_{FCN})$, ${\rm{Im}}({A}_{2})$, ${\rm{Re}}({S}_{2})$, and the siting coefficients $\alpha$, $\beta$, $\gamma$ at each interferometer).

We compute the frequentist probabilities of free model parameters by inverting 100,000 synthetic sets, each consisting of 32 complex tidal amplitudes which we generate by adding a Gaussian random noise (whose standard deviation is given by the formal error of the tidal analysis of real strain data) to each experimental tide (i. e., those given by the tidal analysis of first-differenced strain records). We use a Gaussian random noise since the tidal analysis gives results which are approximately normally-distributed around their “real” values, as a consequence of the almost-white noise spectrum of the first-difference strain sequences^41^, the least squares fitting which tidal analysis is based on, and the large number of analysed data. We assign the same uncertainty to all complex tidal amplitudes in each synthetic data set (which acts as an “experimental” data set for the inversion procedure) and minimize misfit using both the ${L}^{1}$ norm (sum of the magnitudes of the differences between “experimental” and theoretical tides) and ${L}^{2}$ norm (sum of squared magnitudes). As theoretical tides depend on FCN parameters non-linearly, we cannot assume that the misfit function has a single minimum with a quadratic-like shape even when the ${L}^{2}$ norm is considered.

Because of the large number of free parameters, we follow a two-step approach, where the parameter space is divided into two subspaces. The former subspace consists of the four FCN parameters, while the latter consists of the twelve siting coefficients. We rely on forward modelling and minimize misfit by using the downhill simplex algorithm^44^, which requires only function evaluations making almost no special assumptions, in the 4-dimensional FCN subspace. At each sampled point, misfit is computed after estimating the siting coefficients through $\chi$-squared minimization of the distances between “experimental” and theoretical tides; as theoretical tides depend linearly on the siting coefficients, this estimate turns into a matrix inversion. Several tests prove that this approach gives quite similar results to and is much more efficient than minimizing misfit in the 16-dimensional parameter space at once.

### Validation of the inversion procedure

We validate the inversion procedure by using it to invert complex amplitudes of Q_1_, O_1_, P_1_, K_1_, ${\Psi }_{1}$, ${\Phi }_{1}$, N_2_ and M_2_ at each interferometer, obtained (i) computing the theoretical tidal strain components by using the reference^3^ frequency-dependent Love and Shida numbers, ${T}_{FCN}$, and ${Q}_{FCN}$, (ii) combining them by using reasonable local effect coefficients^24,32^, and (iii) assigning them the uncertainties we got from the tidal analysis of the real extension records. Parameters ${T}_{FCN}$, ${\log }_{10}({Q}_{FCN})$, ${\rm{Re}}({A}_{2})$, ${\rm{Im}}({A}_{2})$, and ${\rm{Re}}({S}_{2})$, as well as siting coefficients for each interferometer, are free in the inversions. The same procedure has been repeated after halving ${\Psi }_{1}$ and ${\Phi }_{1}$ uncertainties and/or fixing ${\rm{Re}}({A}_{2})$ to its reference value. Results are shown in Fig. 2b–d. Appropriate tests, whose results are not shown for brevity, demonstrate that fixing $\alpha$, $\beta$, and $\gamma$ does not affect PDFs and correlations appreciably.

### Ocean loading

We compute ocean loading effects on strain tides by means of SPOTL code^45^. SPOTL does not include the most recent ocean models and uses integrated load Green functions computed from tables by^46^ for three old Earth models, namely the Gutenberg-Bullen Earth model A^47^ (GB) and two variants obtained after replacing top 1000 km by the continental shield crust and mantle structure of^48^ and the oceanic crust and mantle structure of^48^. As for ocean models, we add FES2014b^34^ and TPXO9-atlas^49^, released in 2019, to SPOTL, because recent models are more accurate than older ones, mainly in coastal areas. As for Earth models, we add integrated load Green functions computed from tables by^36^ for a PREM^35^ variant obtained after replacing top 40 km by continental crust (cPREM), and tables by^50^ for IASP91^51^. We retain GB for comparison.

We compute ocean loading strain for all the diurnal tidal components included in both FES2014 and TPXO9-atlas, i. e. Q_1_, O_1_, P_1_, and K_1_, along the direction of each interferometer (${\varepsilon }_{xx}$), perpendicularly to it (${\varepsilon }_{yy}$), and as shear strain (${\varepsilon }_{xy}$). We do not use S_1_ in our analysis, because of possible contaminations from environmental effects.

Tides ${\Psi }_{1}$ and ${\Phi }_{1}$ are not included in any ocean model and we estimate their loading effects as follows. It is well known that the relation between the tide generating potential and the complex amplitude of the height of the ocean tides is strongly dependent on frequency as well as on the local and regional conditions that affect ocean dynamics. However, the ratio between the amplitudes of P_1_ and K_1_ ocean loading strain is approximately equal (within 10%) to the ratio of the related potential amplitudes for each interferometer and for each strain component. The only exception is ${\varepsilon }_{yy}$ when $x$ is directed along the GAL16 interferometer, but ${\varepsilon }_{yy}$ gives a small contribution to GAL16 strain because $\beta \ll \alpha$ (Fig. 4b). Phases of P_1_ and K_1_ ocean loading strain differ by few degrees. Moreover, ocean loading contribution to measured deformation in the diurnal tidal band is small for both LNGS and LSC, affecting tidal strain amplitude by less than 5% and phase by at most few degrees (Fig. 1c). We assume that ${\Psi }_{1}$ and ${\Phi }_{1}$ ocean loading strain is related to the tidal potential as for K_1_, but consider that the FCN also affects ocean tides. Thus, we estimate the effects of ${\Psi }_{1}$ and ${\Phi }_{1}$ ocean loading on strain by multiplying K_1_ ocean loading strain by two factors: (i) the ratio of the related potential amplitudes and (ii) the FCN resonant factor, computed inside the inversion procedure using Equations 32 and 33 of^4^.

Although semidiurnal tidal strain is not affected by the FCN, we also compute ocean loading for M_2_ and N_2_, which are used for estimating strain cross-coupling due to siting effects and retrieve the large-scale Earth strain.

Strain load Green functions approximately decline as *r*^−2^ with distance *r* from a point load; thus, ocean loading effects depend on local crust/mantle structure also. Extent of the continental shelf around the two stations (Fig. 1a) suggests that cPREM could be more appropriate than average Earth models. Moreover, PREM is used to compute reference Love and Shida numbers in^3^. Since model parameter PDFs obtained from different ocean and Earth models are very similar to each other (Fig. 4c), for brevity we show most detailed inversion results from FES2014 and cPREM only.

## Data Availability

Datasets are available upon request.

## References

[1] Dehant, V. & Mathews, P. M. *Precession, nutation, and wobble of the Earth* (Cambridge University Press, 2015).

[2] Agnew, D. Earth tides. In Schubert, G. (ed.) *Treatise on Geophysics 2nd Edition*, chap. 3.06, 151–178 (Elsevier, Amsterdam, 2015).

[3] Petit, G. & Luzum, B. *IERS Conventions (2010)* (Verlag des Bundesamts für Kartographie und Geodäsie, 2010).

[4] Mathews PM, Herring TA, Buffet BA (2002). Modeling of nutation-precession: new nutation series for nonrigid Earth and insights into the Earth’s interior. J. Geophys. Res. Solid Earth.

[5] Mathews PM, Buffet BA, Shapiro II (1995). Love numbers for diurnal tides: relation to wobble admittances and resonance expansions. J. Geophys. Res. Solid Earth.

[6] Neuberg J, Hinderer J, Zürn W (1987). Stacking gravity tide observations in central Europe for the retrieval of the complex eigenfrequency of the nearly diurnal free-wobble. Geophys. J. Royal Astron. Soc..

[7] Defraigne P, Dehant V, Hinderer J (1994). Stacking gravity tide measurements and nutation observations in order to determine the complex eigenfrequency of the nearly diurnal free wobble. J. Geophys. Res..

[8] Hinderer J (2000). Are the Free Core Nutation parameters variable in time?. Phys. Earth Planet. Interiors.

[9] Lambert SB, Dehant V (2007). The Earth’s core parameters as seen by the VLBI. Astron. & Astrophys..

[10] Rosat S, Florsch N, Hinderer J, Llubes M (2009). Estimation of the Free Core Nutation parameters from SG data: sensitivity study and comparative analysis using linearized Least-Squares and Bayesian methods. J. Geodyn..

[11] Rosat S, Lambert SB, Gattano C, Calvo M (2017). Earth’s core and inner-core resonances from analysis of VLBI nutation and superconducting gravimeter data. Geophys. J. Int..

[12] Krásná H, Böhm J, Schuh H (2013). Free core nutation observed by VLBI. Astron. & Astrophys..

[13] Malkin Z (2013). Free core nutation and geomagnetic jerks. J. Geodyn..

[14] Belda S, Ferrándiz JM, Heinkelmann R, Nilsson T, Schuh H (2016). Testing a new Free Core Nutation empirical model. J. Geodyn..

[15] Vondrák J, Ron C (2017). New method for determining free core nutation parameters, considering geophysical effects. Astron. & Astrophys..

[16] Nurul Huda I, Lambert S, Bizouard C, Ziegler Y (2020). Nutation terms adjustment to VLBI and implication for the Earth rotation resonance parameters. Geophys. J. Int..

[17] Zhou YH (2016). Estimation of the free core nutation period by the sliding-window complex least-squares fit method. Adv. Space Res..

[18] Cui X, Sun H, Xu J, Zhou J, Chen X (2018). Detection of Free Core Nutation resonance variation in Earth tide from global superconducting gravimeter observations. Earth, Planets Space.

[19] Neuberg, J. & Zürn, W. Investigation of the nearly diurnal resonance using gravity, tilt and strain data simultaneously. In *Proceedings of the tenth international symposium on Earth tides*, 305–311 (1986).

[20] Polzer G, Zürn W, Wenzel HG (1996). NDFW analysis of gravity, strain and tilt data from BFO. Bull. d’Information des Marées Terr..

[21] Zaske J, Zürn W, Wilhelm H (2000). NDFW analysis of borehole water level data from the hot-dry-rock test site Soultz-Sous-Forêts. Bull. d’Information des Marées Terr..

[22] Mukai A, Takemoto S, Yamamoto T (2004). Fluid core resonance revealed from a laser extensometer at the Rokko-Takao station, Kobe, Japan. Geophys. J. Int..

[23] Ping J (2006). Observing long-term FCR variation using Esashi extensometers. J. Geodyn..

[24] Amoruso A, Botta V, Crescentini L (2012). Free Core Resonance parameters from strain data: sensitivity analysis and results from the Gran Sasso (Italy) extensometers. Geophys. J. Int..

[25] Riccardi, U., Boy, J.-P., Hinderer, J., Rosat, S. & Boudin, F. Free Core Nutation parameters from hydrostatic long-base tiltmeter records in Sainte Croix aux Mines (France). In *International Symposium on Earth and Environmental Sciences for Future Generations, Proceedings of the IAG General Assembly*, 171–179, 10.1007/1345-2016-260 (2016).

[26] Milyukov VK (2019). Estimation of Free Core Resonance parameters based on long-term strain observations in the diurnal frequency band. Phys. Solid Earth.

[27] Amoruso A, Crescentini L (2009). The geodetic laser interferometers at Gran Sasso, Italy: recent modifications and correction for local effects. J. Geodyn..

[28] Amoruso A, Crescentini L, Bayo A, Fernández Royo S, Luongo A (2018). Two high-sensitivity laser strainmeters installed in the Canfranc underground laboratory (Spain): Instrument features from 100 to 0.001 mhz. Pure Appl. Geophys..

[29] Harrison JC (1976). Cavity and topographic effects in tilt and strain measurement. J. Geophys. Res..

[30] Meertens CM, Wahr JM (1986). Topographic effect on tilt, strain, and displacement measurements. J. Geophys. Res. Solid Earth.

[31] Park J, Amoruso A, Crescentini L, Boschi E (2008). Long-period toroidal earth free oscillations from the great Sumatra-Andaman earthquake observed by paired laser extensometers in Gran Sasso, Italy. Geophys. J. Int..

[32] Amoruso A, Crescentini L (2016). Nonlinear and minor ocean tides in the Bay of Biscay from the strain tides observed by two geodetic laser strainmeters at Canfranc (Spain). J. Geophys. Res. Ocean..

[33] Díaz, J., Ruíz, M., Crescentini, L., Amoruso, A. & Gallart, J. Seismic monitoring of an alpine mountain river. *J. Geophys. Res. Solid Earth* **119**, 10.1002/2014JB0109557 (2014).

[34] Carrère, L., Lyard, F., Cancet, M., Guillot, A. & Picot, N. FES2014, a new tidal model - validation results and perspectives for improvements. In *Proceedings of the ESA Living Planet Symposium* (2016).

[35] Dziewonski A, Anderson DL (1981). Preliminary reference Earth model. Phys. Earth Planet. Interiors.

[36] Jentzsch, G. Earth tides and Ocean tidal loading. In Wilhelm, H., Zürn, W. & Wenzel, H. G. (eds.) *Tidal Phenomena*, 145–171 (Springer-Verlag, Berlin, 1997).

[37] Métivier L, Conrad CP (2008). Body tides of a convecting, laterally heterogeneous, and aspherical Earth. J. Geophys. Res..

[38] Mathews PM, Buffet BA, Shapiro II (1995). Love numbers for a rotating spheroidal earth: New definitions and numerical values. Geophys. Res. Lett..

[39] Widmer R, Masters G, Gilbert F (1991). Spherically symmetric attenuation within the Earth from normal mode data. Geophys. J. Int..

[40] Crescentini L, Amoruso A, Fiocco G, Visconti G (1997). Installation of a high-sensitivity laser strainmeter in a tunnel in central Italy. Rev. Sci. Instruments.

[41] Amoruso A, Crescentini L, Scarpa R (2000). Removing tidal and atmospheric effects from Earth deformation measurements. Geophys. J. Int..

[42] Baisch S, Bokelmann GHR (1999). Spectral analysis with incomplete time series: an example from seismology. Comput. & Geosci..

[43] Venedikov AP, Arnoso J, Vieira R (2005). New version of program VAV for tidal data processing. Comput. & Geosci..

[44] Nelder JA, Mead R (1965). A simplex method for function minimization. Comput. J..

[45] Agnew, D. C. SPOTL: Some Programs for Ocean-Tide Loading. Tech. Rep., Scripps Institution of Oceanography, San Diego, CA, SIO Technical Report (2014).

[46] Farrell W (1972). Deformation of the Earth by surface load. Rev. Geophys..

[47] Alterman, Z., Jarosch, H. & Pekeris, C. L. Propagation of Rayleigh waves in the earth. *Geophys. J. Int*. **4**, 219–241, DOI: 10.1111/j.1365-246X.1961.tb06815.x (1961).

[48] Harkrider DG (1970). Surface waves in multilayered elastic media (2): higher mode spectra and spectral ratios from point sources in plane-layered Earth models. Bullettin Seismol. Soc. Am..

[49] Egbert GD, Erofeeva SY (2002). Efficient inverse modeling of barotropic ocean tides. J. Atmospheric Ocean. Technol..

[50] Wang H (2012). Load Love numbers and Green’s functions for elastic Earth models PREM, iasp91, ak135, and modified models with refined crustal structure from Crust 2.0. Comput. & Geosci..

[51] Kennett, B. L. N. & Engdahl, E. R. Travel times for global earthquake location and phase identification. *Geophys. J. Int*. **105**, 429–465, DOI: 10.1111/j.1365-246X.1991.tb06724.x (1991).

[52] Tesauro M, Kaban MK, Cloetingh SAPL (2008). EuCRUST-07: A new reference model for the European crust. Geophys. Res. Lett..

[53] Chao BF, Hsieh Y (2015). The Earth’s free core nutation: Formulation of dynamics and estimation of eigenperiod from the very-long-baseline interferometry data. Earth Planet. Sci. Lett..

